# Medicinal Foods, YT and RH Combination, Suppress Cigarette Smoke-Induced Inflammation and Oxidative Stress by Inhibiting NF-*κ*B/ERK Signaling Pathways

**DOI:** 10.1155/2022/4525758

**Published:** 2022-03-14

**Authors:** Yuanyuan Li, Lan Wang, Jieping Luo, Yuqin Chen, Wenju Lu

**Affiliations:** ^1^Guangzhou Medical University, Guangzhou, China; ^2^Guangzhou Institute of Respiratory Health, The 1st Affiliated Hospital of Guangzhou Medical University, Guangzhou, China

## Abstract

**Background:**

Cigarette smoke is a risk factor for Chronic Obstructive Pulmonary Disease (COPD). Given the lack of COPD curative treatment, dietary management for COPD patients has become important. This study investigated whether the medicinal foods (YT and RH) could suppress cigarette smoke exposure-induced inflammation and oxidative stress.

**Methods:**

Chronic pulmonary inflammation in male C57 mice was induced by a 4-week exposure to cigarette smoke (CS). The medicinal foods YT and RH were orally administered 1 week prior to CS exposure. The protective effects were assessed by measuring the pulmonary function and histopathological evaluations. Inflammatory cell numbers and cytokines levels in BALF and blood serum were analyzed by enzyme-linked immunosorbent assay (ELISA). Malondialdehyde (MDA) and superoxide dismutase (SOD) levels of the lung were analyzed. Furthermore, the levels of phosphorylated ERK and NF-*κ*B in both the mice lungs and RAW264.7 cells were also detected.

**Results:**

YT and RH combination (YT + RH) significantly improved pulmonary function and suppressed the inflammation, including cell number and cytokines in BALF relative to the CS group; histological examination revealed protective effects of YT + RH in the lungs of mice exposed to CS. Moreover, the MDA level in the lung of the YT + RH group of mice was lower, the SOD activity was higher, and *in vitro* treatment of YT and RH combination attenuated reactive oxygen species (ROS) expression in mouse macrophage RAW264.7 cells stimulated with cigarette smoke (CSE). YT + RH combination significantly reduced the expression of pNF-*κ*B and pERK in the lung tissues and macrophage stimulated with CSE.

**Conclusions:**

YT and RH combination attenuates cigarette smoke-induced inflammation and oxidative stress through inhibition of the NF-*κ*B/ERK signaling pathway.

## 1. Background

Chronic Obstructive Pulmonary Disease (COPD) is characterized by progressive airflow limitation, chronic pulmonary inflammation, and emphysema. The study on the prevalence for 2010 across world regions showed that the overall prevalence in men aged 30 years or more was 14.3% (95% CI 13.3%–15.3%) compared to 7.6% (95% CI 7.0%–8.2%) in women [1]. In addition, COPD is predicted to become the third leading cause of morbidity and mortality worldwide [2–4]. COPD has been a major public health problem and will remain a challenge within the twenty-first century because of its high prevalence, morbidity, and mortality. Therefore, developing new methods to prevent and manage COPD is essential, with important social and economic significance.

Cigarette smoke (CS) is a critical risk factor for COPD [5, 6]. Besides the induction of consistent airway inflammatory responses, CS induces the production of enormous amounts of ROS, which are mainly released from activated cells, including macrophages, neutrophils, or structural cells like epithelial cells during COPD [7, 8]. Oxidative stress occurs when ROS is produced beyond the antioxidant capacity and damages the cellular components, such as DNA, lipids, and proteins. Such damage could result in lung cell death, degradation of extracellular matrix, and loss of alveolar unit [9, 10]. Oxidative stress also induces inflammatory response in the airway, leading to bronchial wall remodeling, mucosal thickening, and mucus hypersecretion [10]. A short-term protective effect of antioxidants on lung function was observed in two intervention studies in subjects with high exposure to oxidative air pollution under natural conditions [11].

Based on other previous studies, consumption of certain fruits, vegetables, whole grains, and fish may prevent COPD, while consumption of processed foods may increase the prevalence of COPD [12, 13]. A healthy diet has protective effects on the biological processes involved in lung function, disease development, and outcomes [9].

The ingredients of YT and RH are extracted from Chinese medicinal foods; YT consists of *Poria cocos*, *Dictyophora indusia*, *Lentinus edodes*, *Tremella fuciformis*, and *Astragalus membranaceus*, the ingredients in which has been reported to exert antioxidative stress *in vivo* and *in vitro* [14–17]. RH is made of honey, *Saccharum sinense* Roxb., *Ficus carica*, *Imperata cylindrica* rhizomes, *Eleocharis tuberosa*, and lily bulb. According to Chinese traditional medicine theory, these ingredients are helpful to the respiratory system. However, whether YT or RH can reduce COPD pulmonary inflammation and oxidative stress is still unclear.

In this study, we hypothesized that YT or RH elicits its protective effects by suppressing pulmonary inflammation and oxidative stress. To test the hypothesis, we administrated the YT and/or RH to CS-exposed mice and examined the protection effect by investigating the mice pulmonary function, histological changes in lung, and oxidative stress levels. Moreover, potential underlying molecular mechanisms are also explored.

## 2. Materials and Methods

### 2.1. Chemicals and Reagents

YT and RH were sourced from Infinitus (China) Co., Ltd. (Guangzhou, China). The cigarettes (YeShu Label: tar 11 mg/cigarette, nicotine 1 mg/cigarette, and CO yield 13 mg/cigarette) were purchased from China Tobacco Guangdong Industrial Co., Ltd. (Guangzhou, China). SOD and MDA assay kits were purchased from Nanjing Jiancheng Bioengineering Institute (Nanjing, China). KC and MCP1 ELISA kits were purchased from R&D (Minneapolis, USA). Antibodies against pNF-*κ*B p65 and HO-1 were purchased from Abcam Biotechnology (Cambridge, USA); antibodies against pERK, ERK and GAPDH were purchased from Cell Signaling Technology (MA, USA); H2DCFDA were purchased from Thermo Fisher Scientific (MA, USA); the HRP-labeled goat anti-rabbit/mouse IgG (*H* + *L*) were purchased from Abcam Biotechnology (Cambridge, USA). The polyvinylidene fluoride (PVDF) membranes were obtained from Millipore Corporation (Billerica, USA). ECL-Plus detection kit probe was purchased from Tanon Science & Technology Co., Ltd. (Shanghai, China). All cell culture reagents were purchased from Gibco (Carlsbad, CA), and the rest were purchased from GBCBIO Technologies Inc. (Guangzhou, China) unless stated otherwise.

### 2.2. Preparation of YT Dry Powder and RH

*Wolfiporia extensa*, *Phallus indusiatus*, *Lentinula edodes*, *Astragalus*, and *Tremella fuciformis* were prepared and mixed at a ratio of 5 : 5 : 2.5 : 2.5 : 1.7, and a two-time extraction was performed. For the first time, add 15× volume of water and extract for 2 hours at 100°C. For the second time, add 12× volume of water and extract for 1 hour at 100°C. Then, the two extracts were combined, filtered, and vacuum-compressed. The concentrated extraction was cooled down in a cold room (−2°C ± 2°C) for 24 hours and then centrifuged and spray dried to obtain the dry powder (containing polysaccharide content ≥5%) with an extraction rate of 25%–30%.

RH was prepared by a mix of honey (17 g), lily concentrate (2 g), fig juice concentrate (2 g), *Perotis indica* root concentrate (1 g), and *Eleocharis dulcis* concentrate (1 g). The honey was purchased from Guangxi Wuzhou Tianmijia Bee Industry Co., Ltd. (Catalog: 30170057), Guangxi, China. The lily concentrate, fig juice concentrate, *Perotis indica* root concentrate, and *Eleocharis dulcis* concentrate were prepared by water extraction-centrifugation-concentration to 1 g concentrated solution corresponding to 1 g medicinal materials, with solid contents of 20%, 40%, 20%, and 6%, respectively.

### 2.3. Animals and Treatment

Male C57/BL/6 mice (6-7 weeks) were sourced from Beijing Vital River Laboratory Animal Technology Co., Ltd. (Beijing, China) and housed in the specific pathogen-free (SPF) facility at a controlled temperature of 25°C with a 12 h photoperiod. The animal experimental protocol was approved by the Ethics Committee of Huamiao Biotechnology Co., Ltd. The mice were randomly divided into six groups according to weight: (1) room air exposure plus saline gavage administration (Blank group); (2) CS exposure plus saline administration (CS group); (3) CS exposure plus YT administration (YT group); (4) CS exposure plus RH administration (RH group); (5) CS exposure plus YT and RH combination administration (YT + RH group); (6) CS exposure plus carbocisteine administration (S-CMC group), *n* = 12 in each group. All protocols were reviewed and approved by the Huamiao Biotechnology Co., Ltd. Animal Care Committee.

Air or cigarette smoke exposure experiments were carried out for six consecutive days for 4 weeks. The cigarette exposure was conducted twice a day with at least a 4 h interval between the sessions. The number of cigarettes and exposure time were gradually increased to the target dose as follows: on the 1st day, mice were exposed to CS of 6 cigarettes for 30 min per session; on the 2nd day, the exposure time with 6 cigarettes increased to 1 h per session; on the 3rd and 4th days, the mice were exposed to CS with 9 cigarettes for 45 minutes per session; on the 5th day, the time of exposure to CS from 9 cigarettes increased to 1 h per session and continued for the rest of the experiment period. Each CS exposure lasted for 2 h with a 20 min break. Mice in the Blank group were restrained for a similar duration with exposure to room air. RH (4 g/kg/day) and YT (0.25 g/kg/day) were orally administered once a day, which began 7 days before CS exposure until the modeling ended. The mice were monitored throughout the smoke exposure procedure, euthanized 24 h after completion of the last exposure.

### 2.4. Pulmonary Function

Pulmonary function of mice was evaluated with the Forced Pulmonary Maneuver System (DSI, CA, USA) according to the manufacturer's protocol. Briefly, mice were anesthetized with 1% pentobarbital sodium (3 ml/kg); then, mice were tracheostomized, intubated, and put in the body chamber of the system. The average breathing frequency of anesthetized animals was forcibly set at 120 breaths/min. Functional residual capacity (FRC) and total lung capacity (TLC) were, respectively, recorded. Initial data were deleted if they were higher than the pressure baseline.

### 2.5. BALF Collection

Lungs were lavaged with 0.6 ml sterile saline solution 6 times via the tracheal tube, the bronchoalveolar lavage fluid (BALF) was centrifuged at 1000 rpm for 5 min followed by supernatant stored at −80°C, and then cell differentials in BALF were evaluated based on morphology with diff-quick staining. At least 200 cells per mouse were counted on the slides in a blinded fashion.

### 2.6. Lung Histology

The mice left lung was fixed overnight in 10% formalin after being inflated by intratracheal instillation of 10% formalin with a 20 cm H_2_O pressure, then the tissue was embedded in paraffin, 4 *μ*m thick sections were made and deparaffinized with xylene and graded ethanol (100%, 95%, 85%, and 75%), and the tissue was stained with hematoxylin and eosin (H&E) followed by two successive 5 min washes in phosphate-buffered saline (PBS). Each slide was examined under a light microscope for alveolar destruction.

### 2.7. Enzyme-Linked Immunosorbent Assay

MCP1 and KC levels in BALF and blood serum were detected with commercial ELISA kits according to the respective manufacturer's instructions.

### 2.8. Measurement of MDA Levels and SOD Activities

The lung tissues of mice were homogenized in cold physiological saline solution, centrifuged at 3500 rpm for 10 min at 4°C, and then the supernatant was collected. Protein contents were determined using a BCA protein assay kit. SOD and MDA levels were measured with the respective kits according to the manufacturer's instructions.

### 2.9. Western Blotting

Mouse lung tissues and RAW 264.7 cells were homogenized in RIPA lysis buffer containing a 1% protease inhibitor cocktail (Sigma-Aldrich, MO, USA), and 30 *μ*g protein was separated by 10% sodium dodecyl sulfate-polyacrylamide gel electrophoresis (SDS-PAGE) (Bio-Rad Laboratories, CA, USA). The proteins were transferred to PVDF membranes and then incubated with a blocking solution (5% skim milk in TBST) for 1 h at room temperature; after being washed three times with TBST, the membranes were incubated overnight with specific antibodies against NF-*κ*B p65 (1 : 1000), HO1(1 : 5000), pERK, ERK, and GAPDH (1 : 3000), respectively, at 4°C. The membranes were thoroughly washed three times with TBST before incubation with horseradish peroxidase- (HRP-) conjugated antibodies for 1 h at room temperature. After another TBST wash, the membranes were exposed to enhanced chemiluminescence (ECL) detection. Western blot image was obtained from the Tanon 5200 Chemiluminescence Imaging System (Shanghai Tanon Science & Technology, Shanghai, China).

### 2.10. Immunohistochemistry (IHC) Assay

Paraffin-embedded mouse lung section was dewaxed as described in the “Lung Histology” section. Antigen retrieval was performed in 0.01 M sodium citrate buffer (pH 6.0); after microwaved at 100°C for 15 min, the slides were washed in PBS three times; subsequently, the sections were incubated at 37°C for 30 min in 3% hydrogen peroxide in methanol to block endogenous peroxidase activity followed by washing in PBS for 3 times; the slides were blocked at 37°C for 60 min with 5% bovine serum albumin (BSA) and then stained with specific antibodies for p-NF-*κ*B (1 : 200) at 4°C in a humidified chamber overnight. The slides were washed in PBS 3 times before being incubated with a second antibody at room temperature for 60 min; then, the slides were stained with diaminobenzidine (DAB). All sections were counterstained with hematoxylin and examined under an Olympus light microscope. Dark brown color signified positive immunostaining for a particular antigen expression. Negative controls were prepared by IgG for the primary antibodies.

### 2.11. Immunofluorescence (IF) Assay

The cells were fixed with 4% paraformaldehyde and then permeabilized in 0.1%Triton X-100 and 5% BSA for 30 min. Primary antibodies diluted in PBS were incubated overnight at 4°C; the cells were washed three times with PBS and stained with a secondary antibody for 1 h at room temperature. After washing, the samples were covered with PBS containing 50% glycerol. Images were obtained using microscopes.

### 2.12. Preparation of CSE

To prepare CSE stock solution, smoke from 2 cigarettes was passed through 10 ml serum-free Dulbecco's modified Eagle's medium and sterilized via filtration through a 0.22 um filter. The CSE stock solution (100%) was diluted to the desired concentrations in the experiment.

### 2.13. Cell Culture

RAW264.7 cells sourced from the Cell Bank of the Chinese Academy of Science (Shanghai, China) were cultured in DMEM (10% FBS, 100 U/mL penicillin, and 100 *μ*g/mL streptomycin) in a humidified incubator with 5% CO_2_ at 37°C. RAW264.7 cells were seeded in 6-well plates with a 60–70% concentration and then treated with 3% CSE plus YT, RH, and YT + RH, respectively. Cells were washed three times with cold PBS 24 h after the treatments, and cell lysates were harvested for Western blot analysis.

### 2.14. Measurement of ROS

Total ROS production in RAW264.7 cells was determined by the DCFDA fluorescence microscopy method as follows: RAW264.7 (approximately 70% concentration in 6-well plates) cells were incubated with 10 mM DCFDA for 30 minutes at 37°C. Fluorescence of oxidized DCFDA and the index of ROS formation were measured with a fluorescence microscope excitation and emission set at 490 and 530 nm, respectively. CSE-induced ROS formation in cells was quantified with IPP.

### 2.15. Statistical Analysis

All results are described as the means ± standard deviation (SD). Statistical difference among the experimental groups was detected by the one-way ANOVA analysis with IBM SPSS v20.0. Statistical significance was set at *P* < 0.05.

## 3. Results

### 3.1. YT + RH Improves Pulmonary Function and Alleviates Emphysema in CS-Exposed Mice

**Figure 1:**
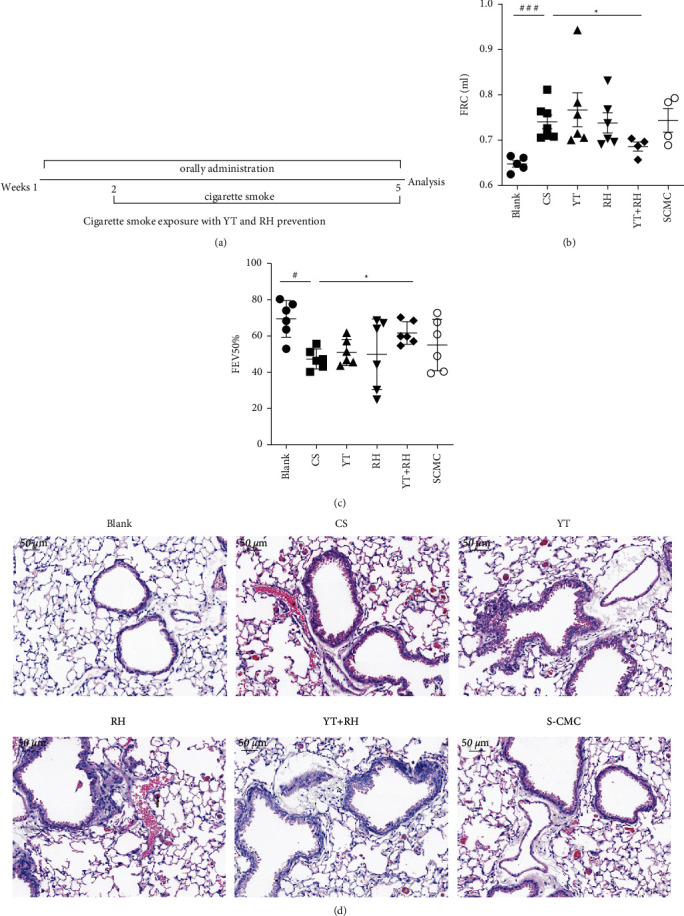
YT + RH improves pulmonary function and emphysema in CS-exposed mice. (a) Mice were exposed to CS for 4 weeks and pretreated. (b, c) The pulmonary function of mice was measured. (d) Pathological changes in the lungs of mice (H&E stained). Scale bar = 50 *μ*m, *n* = 6 in each group. The values are presented as the mean ± SD. ^#^*P*＜0.05 and ^###^*P* ＜ 0.001 versus the Blank group;  ^*∗*^*P* ＜ 0.05 versus the CS group. YT + RH reduces CS-induced inflammation in mice.

Mice were exposed to CS for 4 weeks (Figure 1(a)). Compared with mice of the Blank group, CS-exposed mice presented declined weight and pulmonary function with increased FRC and decreased FEV50%; the pulmonary function of YT + RH group mice was significantly improved, as demonstrated by decreased FRC and increased FEV50% (Figures 1(b) and 1(c)). Pathologic alterations were examined after H&E staining, as shown in Figure 1(d). Four-week CS exposure induced significant emphysema. Such changes were significantly alleviated by YT + RH treatment.

**Figure 2:**
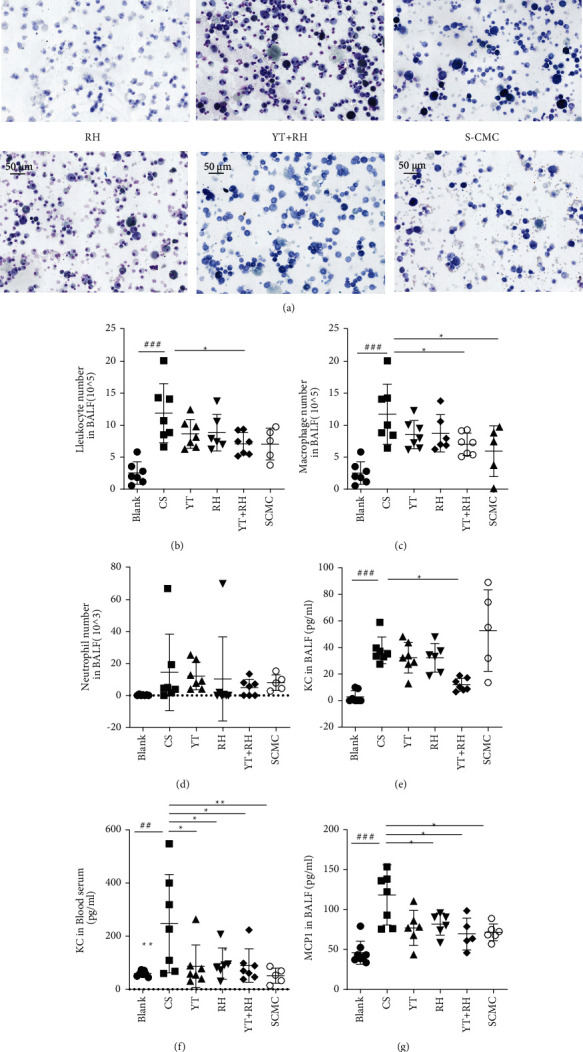
YT + RH reduces CS-induced inflammation in mice. (a) The inflammation cells differentials in BALF were stained with diff-quick staining. (b–d) The total counts of cells, macrophages, and neutrophils from the BALF were counted. (e–j) Inflammatory cytokines were detected by ELISA. *n* = 7 in the Blank group, CS group, YT group, and YT + RH group, *n* = 6 in RH group, and *n* = 5 in SCMC group. The values are presented as the mean ± SD. ^##^*P*＜0.01 and ^###^*P*＜0.001 versus the Blank group;  ^*∗*^*P* ＜ 0.05,  ^*∗∗*^*P* ＜ 0.01, and  ^*∗∗∗*^*P* ＜ 0.001 versus the CS group.

Mice exposed to CS for 4 weeks exhibited typical inflammation, in consistent with previous findings [18, 19]. We measured the cell counts and differentiation in BALF to assess the effects of YT and RH on the CS-induced pulmonary inflammation (Figure 2(a)). CS significantly increased the number of inflammatory cells, including the cell counts of total cells, macrophages, and neutrophils. YT + RH reduced inflammatory cell recruitment in the lung (Figures 2(b)–2(d)). Evaluation of the effects of YT + RH on cytokine secretion in CS-treated mice revealed that YT + RH reduced CS-induced increase of KC and MCP-1 in the BALF (Figure 2(e) and 2(g)) and KC in the blood serum (Figure 2(f)). These results suggest that the administration of YT + RH could attenuate CS-induced airway inflammation.

### 3.2. YT + RH Attenuates CS-Induced Oxidative Stress in Mice

**Figure 3:**
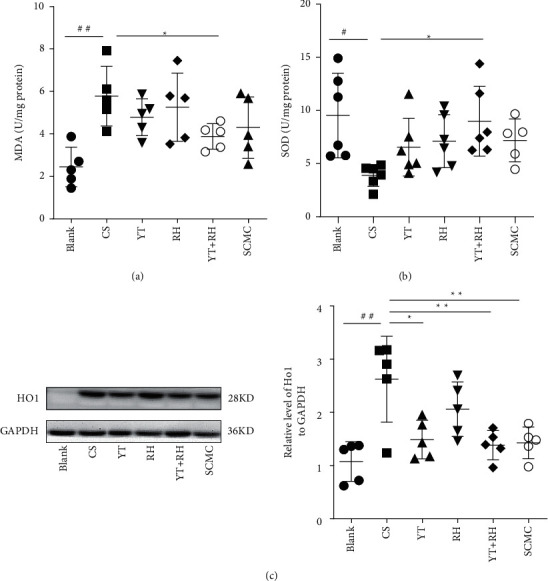
YT + RH attenuates CS-induced oxidative stress in mice. (a, b) SOD activity and MDA levels in the lungs of mice MDA and SOD activity of lungs were measured. (c) HO1 expression of the lung was measured by WB, *n* = 5 in each group. ^##^*P* ＜ 0.01 and ^###^*P* ＜ 0.001 versus the Blank group;  ^*∗*^*P* ＜ 0.05, ^*∗∗*^*P* ＜ 0.01, and  ^*∗∗∗*^*P* ＜ 0.001 versus the CS group.

CS causes oxidative stress in the mouse lungs as demonstrated by the increased and decreased MDA levels and SOD activities, respectively. YT + RH treatment significantly attenuated MDA levels and decreased SOD activities (Figures 3(a) and 3(b)). Moreover, the Western blotting analysis showed that the protein levels of HO-1 were significantly enhanced in the lungs of CS-exposed mice; treatment with YT + RH reduced protein levels of HO-1, indicating the inhibitory effect of YT + RH on oxidative stress.

### 3.3. YT + RH Treatment Attenuates CSE-Induced Oxidative Stress in Macrophages

**Figure 4:**
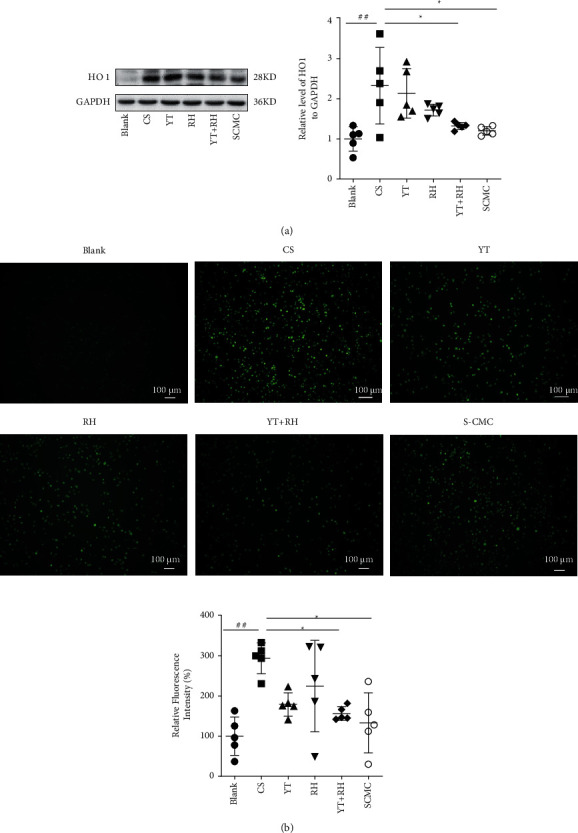
YT + RH attenuates CSE-induced oxidative stress in macrophages. (a) HO1 expression of the lung was measured by WB. (b) ROS production in RAW267.4 cell was analyzed by fluorescence microscopy. The values are presented as the mean ± SD of five individual experiments. ^##^*P*＜0.01 and ^###^*P*＜0.001 versus the Blank group;  ^*∗*^*P*＜0.05, ^*∗∗*^*P*＜0.01, and  ^*∗∗∗*^*P*＜0.001 versus the CS group.

CSE induces oxidative stress in macrophages [20]. We also investigated the effects of YT + RH treatment on CS-induced oxidative stress in macrophages. Western blotting analysis revealed a significant increase in the expression level of HO-1 in the CSE-induced macrophage; YT + RH significantly reduced the HO-1 expression levels in the CS-stimulated mice (Figure 4(a)). Given that oxidation reactions induced by ROS are regarded as a trigger of the oxidative stress, we examined the effects of YT + RH on intracellular ROS production in RAW 264.7 cells. As shown in Figure 4(a), CSE significantly increased ROS levels of macrophages, which were significantly inhibited by YT + RH (Figure 4(b)).

### 3.4. YT + RH Treatment Inhibits CS-Induced Activation of pERK and pNF-*κ*B *In Vivo*

**Figure 5:**
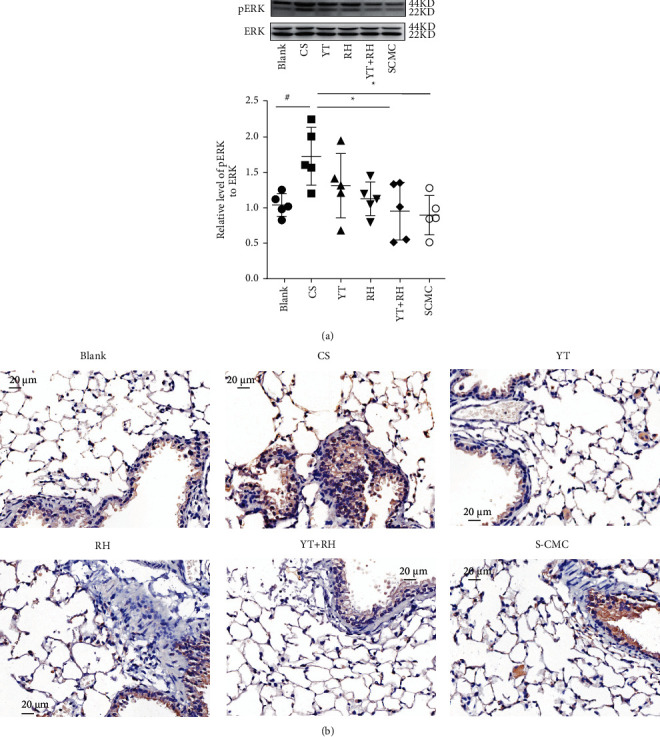
YT + RH treatment inhibits CS-induced activation of pERK1/2 and pNF-*κ*B *in vivo*. (a) The expression of pNF-*κ*B in the lungs was detected by immunohistochemical experiments. (b) The expression of phosphorylation ERK of the lung was detected by WB. The values are presented as mean ± SD of five individual experiments. ^##^*P* ＜ 0.01 and ^###^*P* ＜ 0.001 versus the Blank group;  ^*∗*^*P* ＜ 0.05, ^*∗∗*^*P* ＜ 0.01, and  ^*∗∗∗*^*P* ＜ 0.001 versus the CS group.

Given that MAPKs and NF-*κ*B signaling pathways play important roles in the regulation of CS-induced inflammation and oxidative stress [21–23], we investigated the effects of YT + RH on the phosphorylation of ERK and NF-*κ*B. As demonstrated by the Western blot (Figure 5(b)) and immunohistochemical (Figure 5(a)) analyses, the pERK and pNF-*κ*B were remarkably increased in the CS-exposed mouse lungs. YT + RH treatment significantly reduced these levels.

### 3.5. YT + RH Treatment Inhibits CS-Induced Activation of NF-*κ*B *In Vitro*

**Figure 6:**
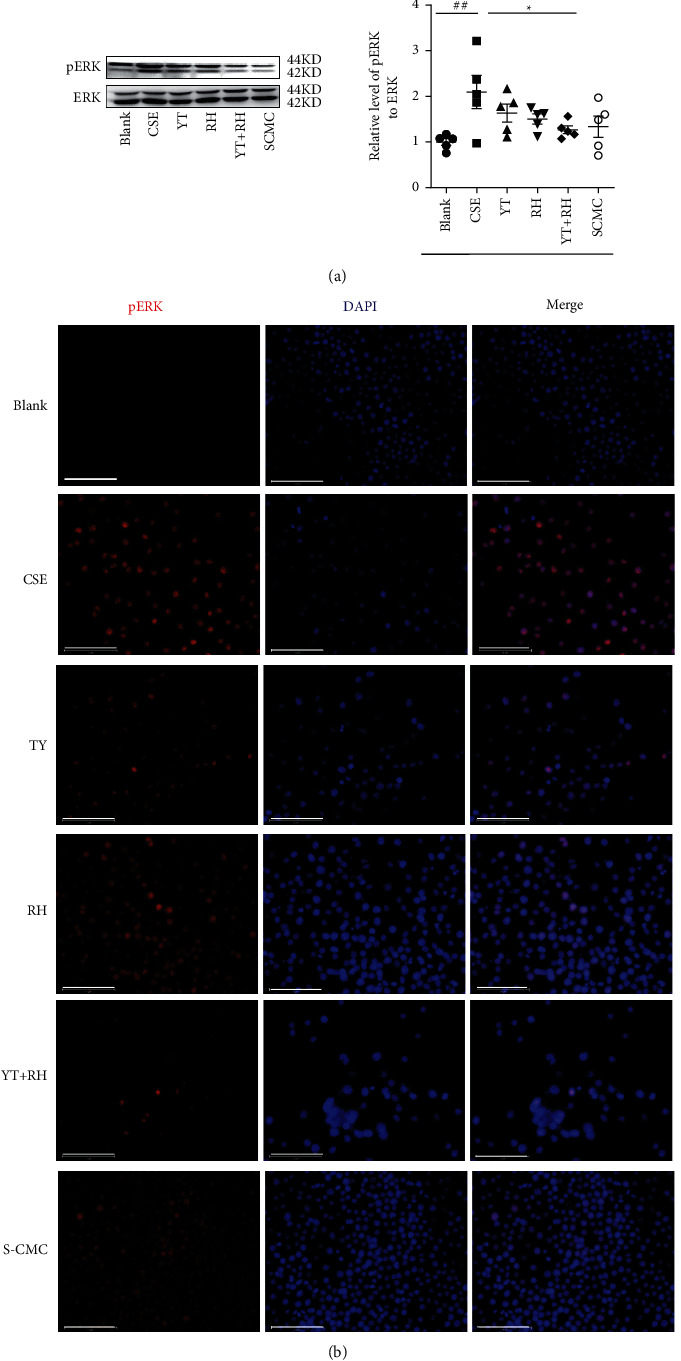
YT + RH treatment inhibits CS-induced activation of pNF-*κ*B *in vitro*. (a) The phosphorylation NF-*κ*B expression of cell was detected by WB. (b) The phosphorylation NF-*κ*B expression of cell was detected by IF. The values are presented as the mean ± SD of five individual experiments. ^#^*P* ＜ 0.05 versus the Blank group;  ^*∗*^*P* ＜ 0.05 versus the CS group.

The suppression effects of YT + RH on pNF-*κ*B signaling pathways were validated in RAW 264.7 cells. Western blot analysis showed that CSE stimulation significantly increased the phosphorylation of NF-*κ*B (Figure 6(a). Immunofluorescence of RAW 264.7 cells stimulated with CSE showed increased pNF-*κ*B levels (Figure 6(b)). Collectively, these data suggest that the inactivation of NF-*κ*B signaling pathways contributes to the protective effects of YT + RH on the CS-induced inflammation and oxidative stress.

### 3.6. YT + RH Treatment Inhibits CS-Induced Activation of pERK *In Vitro*

**Figure 7:**
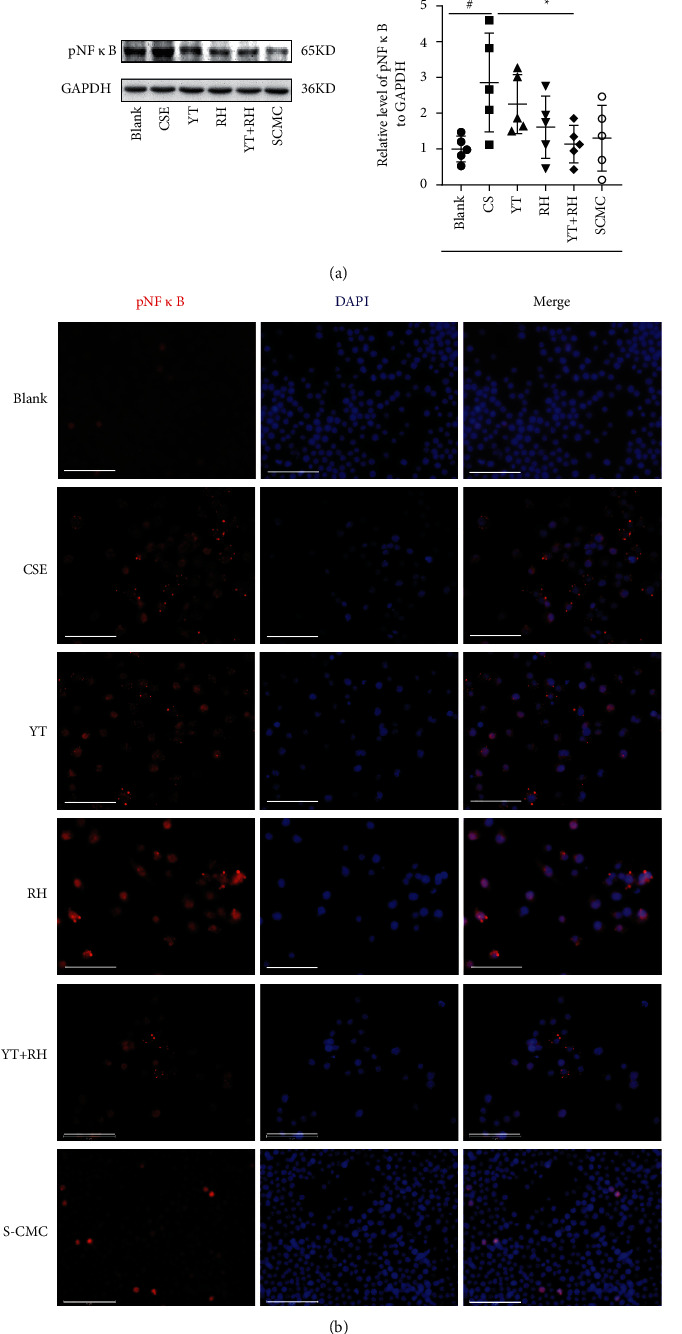
YT + RH treatment inhibits CS-induced phosphorylation of ERK *in vitro*. (a) The phosphorylation ERK expression of cell was detected by WB. (b) The phosphorylated ERK expression of cell was detected by IF. The values are presented as the mean ± SD of five individual experiments. ^#^*P* ＜ 0.05 versus the Blank group;  ^*∗*^*P* ＜ 0.05 versus the CS group.

As shown in Figure 7(a), the levels of phosphorylated ERK were significantly increased in RAW264.7 cells after stimulation of CSE, which can be attenuated by treatment of YT + RH. Immunofluorescence of RAW 264.7 cells stimulated with CSE showed increased ERK levels (Figure 7(b)). Altogether, these results suggested that YT + RH protects lung from oxidative stress-induced activation of MAPK signal pathways.

## 4. Discussion

In this study, we demonstrate that the combination of YT and RH intervention (YT + RH) can alleviate CS-induced inflammation and oxidative stress; further, YT + RH successfully inhibited cigarette smoke extract- (CSE-) induced oxidative stress in macrophages. Finally, we demonstrate that the protective effects of YT + RH are associated with the inhibition of CS-induced NF-*κ*B/ERK expression *in vitro* and *in vivo*. These results provide a rationale for the pretreatment of COPD.

COPD is characterized by high incidence mortality and disability rate, thereby posing a serious threat to human health. Presently symptomatic strategies such as anti-inflammatory, antispasmodic, and antiasthmatic are widely used in the treatment of COPD. However, given that there are no effective treatments to inhibit chronic inflammation in COPD, prevention treatment may be a viable strategy. Diet may contribute to antioxidant/oxidant and inflammatory status in COPD patients. Compared with healthy individuals, COPD subjects have diets with lower fruit, vegetable intake, and poorer antioxidant content [13], correlating with the impaired lung function and risk of developing COPD [24, 25]. YT and RH are medicinal foods that have anti-inflammation and antioxidative properties; therefore, we investigated the potential of these properties. This is the first study to report that the YT and RH combination can inhibit cigarette smoke-induced lung inflammation and oxidative stress through a mechanism that involves the downregulation of inflammatory cytokines NF-*κ*B.

Previous studies demonstrated pachymic acid, a lanostane-type triterpenoid from *Poria cocos*, has been reported to reduce apoptosis by activating ROS-dependent JNK and ER stress pathways in lung cancer cells [17]. *Dictyophora indusial*, *Lentinus edodes*, and *Tremella fuciformis* belong to different species of edible and/or medicinal mushrooms, which possess antioxidant activity due to their bioactive compounds such as polyphenols, polysaccharides, vitamins, carotenoids, and minerals [16]. In addition, they have antiobesity, antidiabetes, anticancer, and antibiotic properties [15], and *Dictyophora indusial* polysaccharides have an immune-stimulatory effect [26]. For instance, cytokines (IL-1*β*, IL-6, and TNF-*α*), NO synthase, and NF-*κ*B of the RAW264.7 cells were upregulated upon treatment with *Dictyophora indusial* polysaccharides [14]. The *β*-glucan from *Pleurotus Ostreatus*, which is another species of mushroom, decreased the incidence and duration of bacterial exacerbations in patients with COPD [27]. Moreover, *Astragalus membranaceus* extract reduced the inflammatory response induced by lipopolysaccharide from *E*. *coli* (LPS), reduced ROS release, and increased nuclear factor (erythroid-derived 2)-like 2 (Nrf2) activation [28, 29].

Honey has a protective effect against oxidative stress and inflammatory response [30]. *Ficus carica* fruits manifested its anticancer properties via inhibiting proliferation, apoptosis, and necrosis of Huh7it cells [31]. *Ficus carica* has emerged as an ideal source of traditional medicine and food for the treatment of various ailments such as anemia, cancer, diabetes, leprosy, liver diseases, paralysis, skin diseases, and ulcers [32]. Flavonoid compounds from *Eleocharis tuberosa* peel have also shown antitumor, antioxidant, and nitrite scavenging effects [33]. Polysaccharides of lily bulb ameliorated menopause-like behavior in ovariectomized mice [34].

There is evidence that increased oxidant stress and inflammation with cigarette smoke injury lung and oxidative stress marks, such as increased MDA and decreased SOD activities, were observed in this study, and YT and RH combination significantly inhibited CS-induced increased MDA and decreased SOD, suggesting that YT and RH combination could attenuate CS-induced oxidative stress. Nuclear factor NF-*κ*B (prooxidative) is one of the redox-sensitive transcription factors that coordinate the inflammatory response to cigarette smoke. The activation of these transcription factors is often evident in both the lung and extrapulmonary tissues [35]. In this study, we detected whether YT and RH protected against CS-induced oxidative stress and inflammation by activating NF-*κ*B. Results showed YT and RH combination could downregulate the phosphorylation of both NF-*κ*B and ERK. However, after administration of RH or YT, respectively, the treatment group exhibited that lung inflammation and oxidative stress were not significantly reversed; we suspect that the administration dose may be too low to allow RH or YT pretreatment to be efficacious.

Chronic CS exposure is associated with emphysema and airway remodeling in COPD patients. In this study, we used a CS-induced mouse model to investigate the effects of YT and RH on lung inflammation. Four weeks of cigarette smoke exposure was enough to induce inflammation in this model, in consistent with previous studies [36]. The mouse model of cigarette smoke exposure was designed to evaluate the degree of inhibition of cigarette smoke-induced lung inflammation, but not the emphysema observed in chronic mouse cigarette smoke exposure models [36, 37]. Human bronchial epithelium cell lines exposure to cigarette smoke augmented the release of neutrophil chemoattractant IL-8 and monocyte chemoattractant protein 1 (MCP-1) [38, 39]. Cigarette smoke induced the production and accumulation of burden oxidants in the respiratory tract [20, 40]. Additionally, oxidative stress in the lung can trigger inflammation induced by CS [19]. Long-term use of carbocysteine has been documented to reduce exacerbation [41]; hence, it may be considered as an anti-inflammation and antioxidation drug [42, 43]. This informed our choice of carbocysteine as a positive control in this study.

Furthermore, we also analyzed the potential mechanisms of how YT + RH treatment attenuates CS-induced inflammation and oxidative stress. Several studies have previously shown NF-*κ*B to play a pivotal role in COPD inflammation [19]. Moreover, ERK1/2 contributes to CS-induced inflammation by modulating NF-*κ*B DNA-binding activity in A549 cells [42]. We found that YT + RH combination reduced the activation of pERK1/2 and pNF-*κ*B *in vivo* and *in vitro*, indicating that pERK1/2 and pNF-*κ*B signaling may be involved in CS-induced oxidative stress and pathology damage. However, we have not elucidated the causal link mechanism among YT + RH combination treatment, ERK and NF-*κ*B activation, and the disease progression of the CS-induced COPD mice model, which calls for further demonstration.

In conclusion, YT + RH abrogated CS-induced inflammation and oxidative stress. This protective action may be attributable to the anti-inflammatory and antioxidant effects of YT + RH via ERK and NF-*κ*B signaling pathways. This study provides a foundation for further investigation on the protective effects of YT + RH combination on COPD. Although we demonstrate the beneficial effects of YT + RH combination on cigarette smoke exposure in a mouse model, its efficacy in humans should be first assessed in clinical trials.

## Data Availability

The data used to support the study are available from the corresponding author Wenju Lu (e-mail: 2547229277@qq.com).
